# 

**Published:** 1891-02-21

**Authors:** 


					wiNBUKNt'^ ? mmmmm
I for INFANTS T, ** J?, and INVALIDS.
No. 230. vol; IX.] Saturday,
February 21st, 1891.
[One Penny.

				

